# Innovation-facilitating networks create inequality

**DOI:** 10.1098/rspb.2023.2281

**Published:** 2023-11-22

**Authors:** Cody Moser, Paul E. Smaldino

**Affiliations:** ^1^ Department of Cognitive and Information Sciences, University of California, Merced, CA 95343, USA; ^2^ Center for Advanced Study in the Behavioral Sciences, Stanford University, Stanford, CA 94305, USA; ^3^ Santa Fe Institute, Santa Fe, NM 87501, USA

**Keywords:** cultural evolution, inequality, collective intelligence, innovation, networks

## Abstract

Theories of innovation often balance contrasting views that either smart people create smart things or smartly constructed institutions create smart things. While population models have shown factors including population size, connectivity and agent behaviour as crucial for innovation, few have taken the individual-central approach seriously by examining the role individuals play within their groups. To explore how network structures influence not only population-level innovation but also performance among individuals, we studied an agent-based model of the Potions Task, a paradigm developed to test how structure affects a group’s ability to solve a difficult exploration task. We explore how size, connectivity and rates of information sharing in a network influence innovation and how these have an impact on the emergence of inequality in terms of agent contributions. We find, in line with prior work, that population size has a positive effect on innovation, but also find that large and small populations perform similarly *per capita*; that many small groups outperform fewer large groups; that random changes to structure have few effects on innovation in the task; and that the highest performing agents tend to occupy more central positions in the network. Moreover, we show that every network factor which improves innovation leads to a proportional increase in inequality of performance in the network, creating ‘genius effects’ among otherwise ‘dumb’ agents in both idealized and real-world networks.

## 1. Introduction

Why do some populations succeed in building complex innovations while others don’t? Approaches in economics, complex systems, organizational science, and a number of other disciplines implicate a number of factors including cultural norms, ecological affordances, path dependency and luck. Much of this research into innovation has approached the question from one of two perspectives: an agent-positive perspective which focuses on the ability of brilliant or highly skilled individuals in a network to add a great deal of talent to the common pool of resources [1,2], and an agent-negative perspective which focuses on the ability of a network to efficiently transmit information and allow the group as a whole to solve problems [3,4]. In line with the latter perspective, recent work has studied how factors such as population size [5], connectivity [6] and inter-group communication [7] can help individuals explore—and ultimately combine—different ideas to improve collective problem-solving, a phenomenon known as *transient diversity* [8–10].

Prior models have extensively examined tradeoffs between network structures and task completion, such as the finding that decreased connectivity allows for groups to complete more complex tasks and increased connectivity allows for groups to complete simpler tasks [11]; yet the impact that less network connectivity has on the performance of individual agents remains rather opaque. A sufficient understanding of why some individuals provide larger than average contributions to collective performance or of which structures efficiently leverage individual intelligence *per capita* thus remains an underdeveloped aspect of research into collective intelligence. While it is the case that transient diversity increases the ability of a population to improve collective problem-solving, the presence of such diversity requires that some agents in a population will have better solutions than others [12].

Heterogeneity among better and worse information in the population creates an inequality of performance between agents, which, when linked to network-level performance, can broadly be associated with sociological ideas of the ‘inequality of input’. This is separate from, but loosely related to other forms of inequality of outcome, opportunity or resources [13]. In other words, the maintenance of transient diversity in a fitness landscape necessitates an ‘inequality of success’ between agents in the population, linked to their position in the network and the information they receive from others. Understanding this inequality is important not only because of its importance to collective problem-solving, but also because it may have causal implications for other forms of inequality, including that of wealth, power and opportunity.

We use modelling to show how the relationship between group-level variables and individual performance can help to explain the mismatch between the agent-positive and agent-negative perspectives of innovation, as well as subsequent inequality of performance. Using this framework, we show that patterns similar to Pareto’s ‘law of the vital few’, whereby 20% of individuals perform 80% of the work for an organization [14], can arise as a result of a group’s structure. We also show that not only is it the case that these ‘vital few’ distributions can arise in populations, but that networks which innovate the best also produce the most inequality.

We approach this question by analysing the roles that population size, network connectivity, the diffusion of information by agents and the ability of agents to switch groups play in a model of cumulative innovation. We examine how these factors relate to the speed and quality of innovation, in line with prior work on this topic. We then compare measures of success to the inequality of performance between agents using the Gini coefficient, which has been used widely to assess the heterogeneous contributions of individuals in groups [13,15]. In doing so, we develop an understanding of how factors which bolster innovation are associated with the emergence of apparent differences in agent-level performance.

### (a) Population size

The size of a group has been critically implicated as a factor leading to increased innovation [16–18]. More people bring more ideas. In a mathematical model of social learning, Henrich [5] examined how population size can contribute to both cultural loss and innovation, finding that small populations were vulnerable to cultural loss and larger populations were more likely to innovate. Despite individuals in both populations having the same capacity to learn complex skills from their peers, small populations lacked the variance of skill that large populations possessed and more often drifted below their own mean skill levels; large populations on the other hand drifted past the average learner and continued to innovate. In recent years, a more complex picture of the role of population size on innovation has emerged. Instead of the raw census size of a group being the primary factor bolstering innovation, more critical is a group’s *effective* population size, a broad measure of how extensively diverse a population is [19]. Nevertheless, if connectivity is held constant, increasing the size of the actual population can bolster the effective population size of a group and allow it to find better solutions faster than smaller groups of the same connectivity [20,21]. While increasing innovation, increased population sizes also provide opportunities for more exacerbated inequality, in part due to the increased number of possible comparisons which can be made between individuals. In both network models and real-world populations, increased population sizes and larger networks bring associated decreases in density, which in turn, increase inequality [22–24].

### (b) Connectivity

More structured, or less connected, populations have also been shown to increase effective population sizes by maintaining higher levels of diversity, thereby supporting innovation [6,11,20,25,26]. Reduced connectivity can bolster innovation by either allowing subgroups of a network to work on separate parts of the global problem or by simply altering the flow of information between groups. From an inequality perspective, these mechanisms of restricting information can create heterogeneity in disparate parts of the network, leading to the emergence of inequality. In models of collective problem-solving where problem complexity can be manipulated, fully connected networks perform well on simple tasks while partially connected networks perform much better on complex ones [6,11]. In addition to the structure of the network affecting its connectivity, agent behaviour can also alter this component [10,11]. Examples include variation in agents’ social learning strategies [27], their propensity for risk-taking [21] and their rates of interaction [28]. Individuals may also leave their own group to join others for periods of time to exchange information, as in the case of migration or trade, as found in several extensions of Henrich’s [5] model where increasing movement between groups played a larger role in facilitating innovation than the increase in size of any individual group [7,29,30].

### (c) The Potions Task

**Figure 1.**
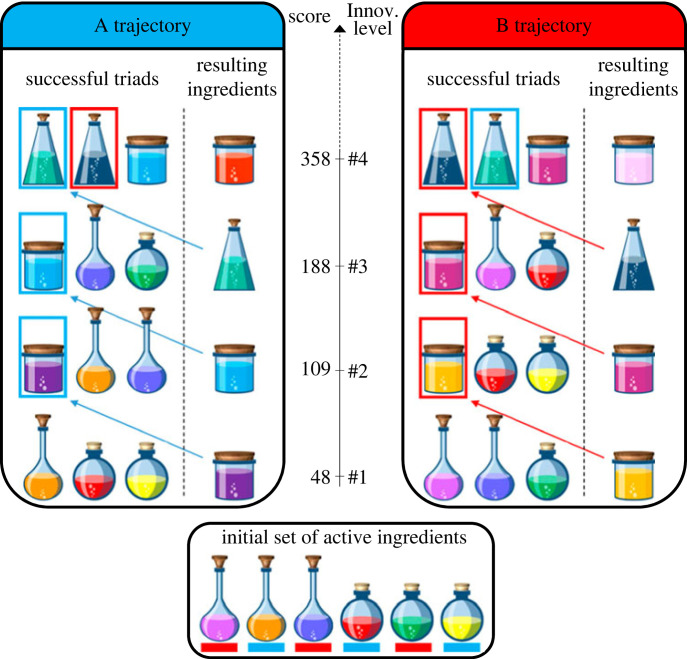
Trajectories in the Potions Task. Combinations are built from a set of six basic ingredients which are then combined to make more complex ingredients. These can themselves be combined to make even more complex ingredients, but the discovery of the two trajectories in the space of innovation depends on the initial combinations made by participants. Each item has a score, shown in the centre column, which reflects how high up on the trajectory it is or its rank. The discovery of the highest-scoring ingredient requires discovering and combining the best solutions from the two respective trajectories. Figure from Derex & Boyd [31].

Derex & Boyd [31] introduced a game called the Potions Task to investigate the link between cumulative innovations, group structure and path dependency using a real-world behavioural experiment. Groups were brought together to play a digital game where each person was provided the same set of six ingredients to mix together into newer ingredients. In the experiment, new ingredients were placed along two separate discovery trajectories, and the most powerful ingredient could only be produced by combining the final ingredients from both trajectories in what the experimenters called a ‘crossover event’ (figure 1). Subjects were placed in one of two group structures: either a ‘fully connected’ group who could mix their own ingredients and see their teammates’ combinations at the end of each round or in ‘partially connected’ groups of dyads that were randomly reassigned partners after several rounds. The authors found that only partially connected groups were able to find the top ingredients in both trajectories and achieve a ‘crossover event’ by combining the two.

This approach was recently adapted into an agent-based model [32] where agents on a real-world hunter–gatherer network were able to combine ingredients in a similar fashion to the previously described experiment. The authors found that their hunter–gatherer networks were able to find powerful crossover innovations much faster than fully connected networks. Two further extensions of this model explored other network architectures, finding that less connected networks consistently outperformed more connected networks while holding population size constant [20] and that core-periphery networks with sparse community structure outperformed less-sparse modular networks [33].

The granular, cumulative and recombinatorial composition of the Potions Task, which was originally used in an experimental context, provides at least two advantages over similar models of collective problem-solving and innovation. First, the game explicitly introduces path dependency to the composition of the task. In the space of possible combinations, there are two trajectories for exploration, and the combination of ingredients at the start guides exploration up one pathway or another (figure 1). Because groups are likely to use new ingredients they discover rather than returning to the initial set, this creates path dependency in the model. A ‘crossover event’ occurs when the highest-performing innovations from each of the two trajectories are first combined to produce an even better innovation. Groups which are able to obtain a crossover event do so because they are able to go backwards in problem space or explore both trajectories simultaneously to overcome this path dependency.

Second, other models of collective problem-solving do not incorporate cumulative innovation, in which multiple discoveries may be recombined to produce a novel innovation. The nature of the Potions Task makes its problem well-defined for asking questions regarding innovation as both a recombinatorial process and one of cumulative advances. Analytically, due to the fact that each agent has a unique inventory of potions of varying scores, modellers can track the individual contributions and payoffs of each individual. We can thus track the progress made by individuals and compare them to others to ask questions about the heterogeneity of work and the impact specific agents have on their networks.

We use the Potions Task to model factors which facilitate innovation in groups and study how they relate to the contributions of individual agents to both group performance and inequality. Groups are tasked with combining triads of ingredients to discover novel innovations. Each agent begins each simulation run with an identical set of six ingredients. At each time step, each agent in a network selects one neighbouring agent at random and the pair combine three ingredients together from their inventories to make a triplet. Agents select which specific item(s) they combine with their partner based on a probability determined by the item’s score (figure 1). If a valid combination is made, the agents in the dyad discover a new item and spread it to their own neighbours with a probability determined by an ‘innovation diffusion’ parameter. Because these new items have a higher score than the items used to create them, they are more likely to be used in subsequent combinations. However, depending on which combinations are made early on, one of two trajectories toward increasingly better potions becomes more likely. This creates path dependency in the model. To examine how switching partners can improve performance at this task, we additionally allow agents to randomly alter one of their links and connect with a new neighbour with a probability based on a ‘change link’ parameter at the end of each step.

The simulation ends either after 1000 steps or when the network has achieved a ‘crossover event’. This is where final innovations in both the A trajectory and the B trajectory are combined, indicating the network has united both paths of exploration. Because each individual holds onto the items they discover and receive from others, we track the maximum innovation scores of each agent’s inventory and calculate a Gini coefficient for the network, which provides a measure of inequality associated with the contributions of individuals to completing the task [15]. A higher Gini coefficient indicates a wider gap in solution quality between the top- and bottom-scoring individuals. A detailed description of our model is given in Material and methods.

## 2. Results

We present our analyses in a piecemeal fashion, asking about the role that population size, connectivity, rates of innovation diffusion, and random link alteration have on cumulative cultural evolution in random networks and the tradeoff between these factors and inequality. We then examine similar factors in connected caveman and several real-world social networks.

### (a) Population size

**Figure 2.**
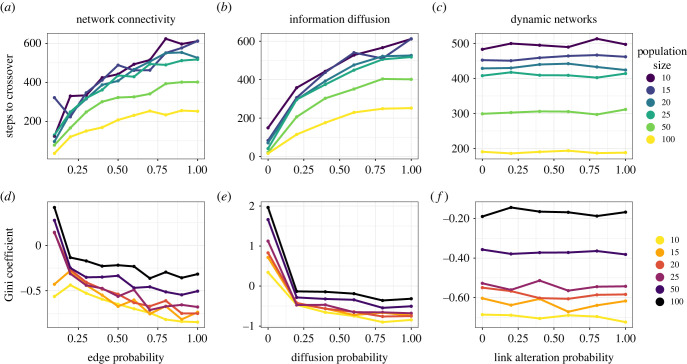
Performance in the Potions Task across three properties of Erdös–Rényi random networks, disaggregated by the measurement used to assess performance, the size of the population and the property being manipulated. (*a*–*c*) Time to a crossover event in the Potions Task as a measure of the number of ‘steps’ in the model, measured by the number of epochs, during which every agent in the population makes a combination with a partner. (*d*–*f*) Normalized Gini coefficients for the same networks in the task. (*a*,*d*) Network connectivity is manipulated by altering the critical edge probability $p=\frac{1}{\left(n-1\right)}$, which is the probability of a possible edge being created when the network is initialized; diffusion was held constant at 1 and random link alteration at 0. (*b*,*e*) Information diffusion is the probability that a given neighbour will receive a new innovation in the Potions Task when it is discovered by the focal agent or their partner (in this case in fully connected networks), a value of 0.5 means that roughly half of the neighbours in this focal network will receive the new innovation, as well; network connectivity was held constant at 1 and random link alteration at 0. (*c*,*f*) Dynamic networks measure the probability that an individual agent will switch one of their current partners to a random agent they are not connected to, an agent with a probability of 0.5 will switch neighbours approximately every other step of the simulation; diffusion was held constant at 1.

**Figure 3.**
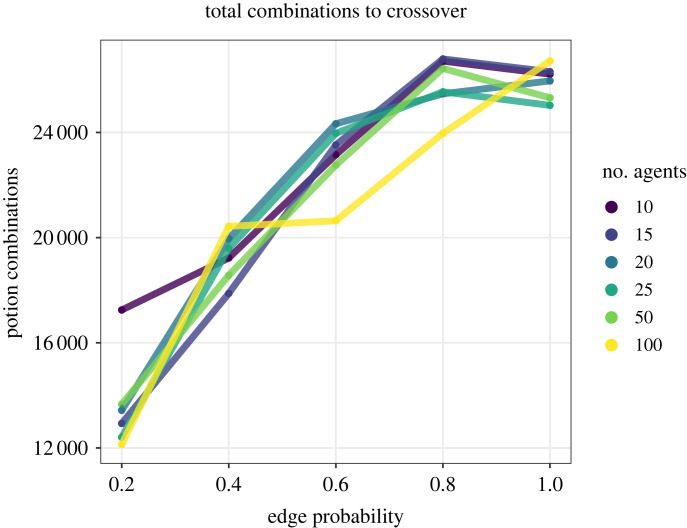
The total number of potion combinations between agents across populations of varying size in random networks. A population of 100 agents completing the task in 100 steps will have made 10 000 combinations, while a population comprised of 10 agents would have to complete the task in 1000 steps to make 10 000 combinations. Diffusion was held constant at 1 and random link alteration at 0.

When measuring by number of steps until a crossover event, large populations outperform smaller ones at all levels of connectivity by obtaining a crossover event faster (figure 2*a*; electronic supplementary material, appendix, figure S1A), as in [20]. When we look at the number of combinations made—that is the number of steps multiplied by the population size—and allow the simulation to run longer for 10 000 steps, we find that networks of all sizes perform similar *per capita* (figure 3), a result that also holds for connected caveman networks (electronic supplementary material, appendix, figure S2). In other words, in the Potions Task, while larger populations outperform smaller ones, it takes roughly the same number of combinations to obtain a crossover, regardless of population scale. This effect persists despite the fact that a large random network with a given edge probability will possess a higher average degree than a smaller random network with the same edge probability.

**Figure 4.**
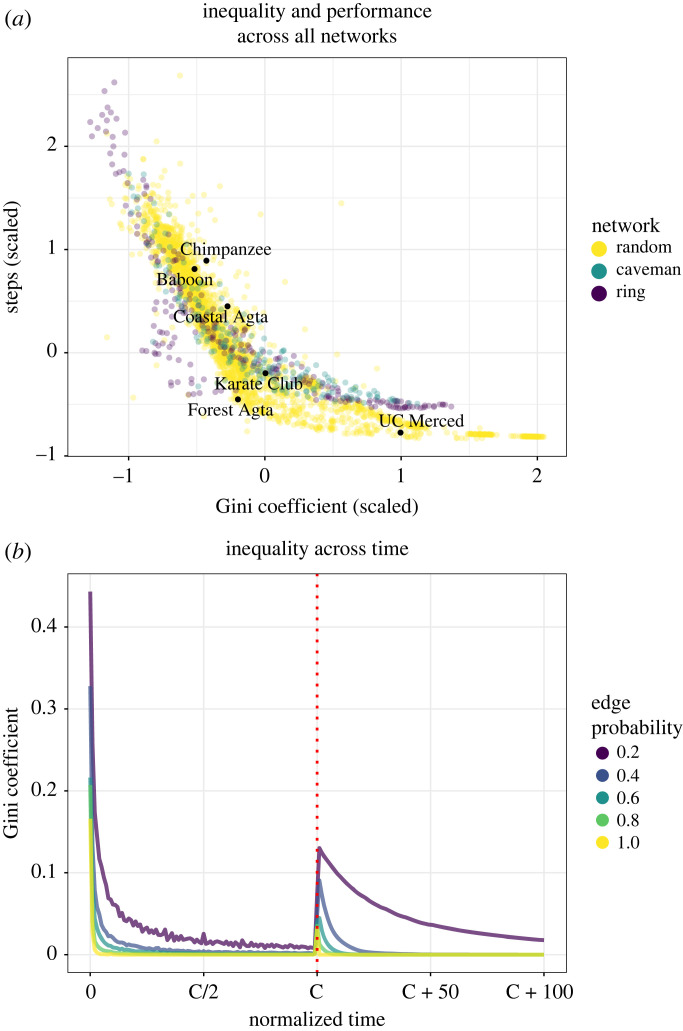
(*a*) The relationship between inequality and performance in the Potions Task for number of steps until crossover. Each point is the average of all runs of a specific parameter combination. (*b*) The persistence of inequality in the Potions Task for a network of 50 agents across varying rates of connectivity. Time until crossover is normalized, after which the simulation continues to run for 100 steps. Diffusion was held constant at 1 and random link alteration at 0.

We find a clear and negative relationship between Gini inequality scores and the size of the network (figure 2*d*; electronic supplementary material, appendix, figure S1D-F). This can be observed in the reversal of the trends between figure 2*a*,*d*, and can also be clearly observed in ring networks where the only manipulated parameter is population size (electronic supplementary material, appendix, figure S3). In each case, the Gini coefficient of the network at the time of crossover is much higher than in larger networks than it is in smaller networks. The relationship between performance and inequality holds true across all parameters (figure 4*a*). These findings are robust to changes to the specific scores we used for the potions. When Gini coefficients were calculated based on the rank scores based on the inventory level of the potions (electronic supplementary material, appendix, figure S4), the same patterns were maintained. Additionally, networks which produced the highest inequality at the time of crossover continued to maintain the same relative levels of inequality for quite some time afterwards, although diffusion in our model’s connected networks requires that inequality always returns to zero in the limit (figure 4*b*). When taking population size into account, we find inequality persists longer in smaller populations (electronic supplementary material, appendix, figure S5). This effect is likely driven by the higher average degree of larger populations, which simultaneously allows for more rapid innovation while diminishing the persistence of inequality through rapid diffusion.

### (b) Connectivity and clustering

We find that less connected networks perform better at all population sizes for a wide range of network architectures, as in [20,31]. This can be seen in figure 2*a* where random networks with fewer connections outperform those with more connections. The results for population connectivity hold regardless of whether one measures success in terms of steps (figure 2*a*) or total combinations (figure 3). These findings support previous work and further generalizes the role that connectivity plays in innovation in populations [11].

We see a similar relationship as with population size with respect to these innovation-bolstering factors and inequality. While there is a positive relationship between connectivity and time until completion of the Potions Task for both measures of performance in random networks, we nevertheless see a stark negative relationship between connectivity and inequality (figure 2*d*). In the simulation, more connected networks, while taking more time to complete the Potions Task, end with a more equitable distribution of outcomes at the point of crossover. In other words, structural heterogeneity of the edges in the network leads to better solutions for the network as a whole at the cost of equality of scores across agents.

#### (i) Connected caveman networks

The connected caveman network divides a population into several strongly connected ‘cliques’ that are weakly connected to one another [34,35]. These networks are created starting with several fully connected cliques arranged on a circle, then choosing one node from each cluster to break one within-cluster link and connect to a parallel node from a neighbouring cluster. This creates a network which maximizes both its sparsity and its clustering. As the ratio of clique count to clique size increases, path length increases and clustering and connectivity decreases (electronic supplementary material, appendix, table S1). Due to these and its cliquish properties, the connected caveman network has been suggested as a potential benchmark for testing questions about collective problem-solving [6,35]. We ran the Potions Task on connected caveman networks, altering the number and size of cliques.

We found that for any given population size, networks which maximize the number of cliques and minimize the size of each clique outperform networks which maximize clique size and minimize clique counts (electronic supplementary material, appendix, figure S6A and table S1). Network statistics can be seen for these networks of equivalent sizes in electronic supplementary material, appendix, table S1, including a comparison of the connected caveman architecture to a ring network of equivalent size. We observe that minimizing the size of cliques and maximizing the number of cliques both decreases connectivity of the network and increases path length, similar to the effect found in random networks when the number of connections are decreased. These results indicate that in the Potions Task a larger number of smaller groups outperform a smaller number of larger groups. These findings strengthen the argument made by a prior model with simpler group structure that populations which exhibit many small groups rather than fewer large groups will tend to be more productive [25].

As with random networks, we find an inverse relationship between the factors which maximize performance in the Potions Task (in these networks, the cliquish nature of the caveman groups) and inequality (electronic supplementary material, appendix, figure S6B). While connected caveman structures can solve the Potions Task with high efficiency, the tradeoff between inequality and performance persists. Networks which have more, but smaller cliques, have more inequality. These effects are additionally exacerbated as the size of the connected caveman network grows and the size of cliques are held constant.

### (c) Diffusion rates

The extent to which agents can share information about good solutions with one another can also affect a population’s ability to solve complex problems. Migliano *et al.* [32] found that when hunter–gatherer groups limited the spread of inventions discovered in the Potions Task only to family members, crossover rates increased. Models of other complex problems have found that decreasing the rate of learning by either making agents less likely to change their priors or by simply decreasing the rate of interaction between them bolster the population’s problem-solving ability [8,25,36]. We test this by altering the probability that any given neighbour of an agent who has made a new discovery receives that agent’s new innovation, such that an agent with a diffusion probability of 0.5 will diffuse the innovation to approximately half of their neighbours. We found that fully connected random networks which limit diffusion outperform those that openly spread information (figure 2*b*).

With respect to inequality, we find a negative relationship between inequality and the diffusion of novel innovations. Separate from the relatively linear observations between diffusion and performance, we observe a nonlinear effect between diffusion and inequality: after a probability of diffusion of 0.2, the negative effects of increasing to higher levels of diffusion are much less than the increase from no levels of diffusion to low levels of diffusion (figure 2*e*). This nonlinear relationship may be partly due to the fully connected nature of these networks. Instead of discovery being clustered in specific subsections of the network, as one would predict in cases of decreased connectivity, the diffused, but slower spread of information allows for the network to preserve transient diversity while nevertheless spreading discovered information across *all* areas of the network, creating fewer clusters of total inequality.

### (d) Dynamic networks

Several models of collective problem-solving have found that dynamically altering networks during computation by severing, adding or changing network links increases performance [7,31,37]. Because agents do not always have access to the information in all parts of the network, alteration of connections allows in some sense for ‘eavesdropping’ by agents. One would predict that due to the propensity for different parts of a network to become stuck on separate trajectories in the Potions Task, the ability to connect to different parts of the network can facilitate crossover events in a population.

We allowed agents to reorganize their network ties by removing one neighbour at random and selecting a new one with a set probability based on a ‘change link’ parameter at the end of each step. We found that in random networks, connection alteration has no effect on either time to crossover or the resulting inequality (figure 2*c*,*f*). Based on the observation that average path lengths scale with $\frac{\log N}{\log K}$ in random networks, leading to particularly short path lengths (less than 2 on average) [38], we also altered dynamic links in random networks, keeping population size constant but altering connectivity (electronic supplementary material, appendix, figure S7), and in connected caveman networks altering clique size and clique number (electronic supplementary material, appendix, figure S8).

We find no effects in our random networks and negative effects in cavemen networks when cliques are kept small, with only small effects otherwise (electronic supplementary material, appendix, figures S7 and S8). In random networks, this is likely due to relatively short path lengths at all levels of connectivity. Conversely, the negative effects observed in connected caveman networks is likely due to an increase in path length and a decrease in cliquishness across the network. While path lengths and connectivity in connected caveman networks are both kept small due to the networks’ cliquishness, dynamic link alteration causes disparate parts of the networks to become connected, increasing the conformity of information across cliques and decreasing the population’s transient diversity.

### (e) Position of high performers

**Figure 5.**
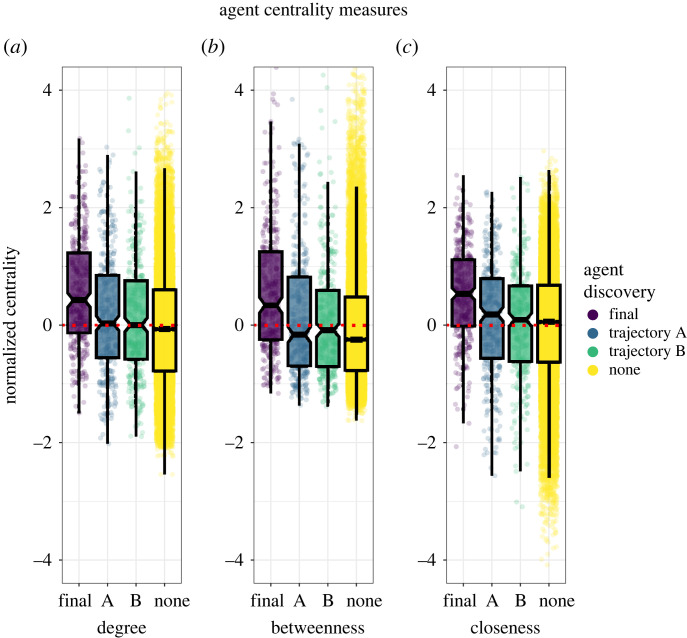
Normalized agent-level metrics of centrality in a random network of 100 agents and 0.05 connectivity, highlighting centrality of first discoverers of the final potion combination, the terminal potions of either trajectories and agents which discovered none of three. (*a*) Degree centrality measures the number of connections a node has in the network. (*b*) Betweenness centrality measures the extent to which a node lies on the shortest path between other nodes in a network. (*c*) Closeness centrality quantifies the average length of the shortest paths from the nodes to all other nodes in the network.

Central to the question of how inequality is manifest in networks is where high-level performers are situated. We examined the network centrality of primary innovators (those who first discover either the solution to the A trajectory or the B trajectory) and those who make the final combination, comparing them to other individuals. We find that primary innovators occupy marginally more central positions than other agents (figure 5). We further find that agents who first obtained final crossovers tended to occupy more central locations in the network than all other agents, including the primary innovators in the A and B trajectories. This was the case for all measures centrality, we considered: degree, betweenness and closeness centralities. In other words, agents who first bring together products of the two trajectories tend to occupy more central positions in the network. In networks with community structure, such nodes may act as ‘bridges’ between otherwise disparate information communities in the network [33].

### (f) Performance in real-world networks

We ran our model on several real-world networks, which allowed us to consider how particular social structures might facilitate or impede innovation in the real world. These included both a chimpanzee and baboon network [39], Zachary’s Karate Club network [40], both hunter–gatherer networks from Migliano *et al.*’s [32] study, and a network representing collaborations among faculty and graduate students in the Department of Cognitive and Information Sciences at the University of California, Merced. The results for this analysis are shown in table 1. It is important to note that the coastal hunter-gatherers and the karate club are of equivalent size to one another, as are the forest hunter–gatherers and the academic department. Yet in a comparison between the karate club and the coastal hunter–gatherers, the karate club performs 48% better and in a comparison between the forest hunter–gatherers and the academic department, the department performs 70% better. As is the case for other architectures, the advantage in these networks is likely due to their decreased connectivity and longer path lengths compared with similar sized counterparts.

**Table 1.** Network statistics for several real-world weighted and unweighted networks. Each network was run for 1000 iterations.

network	N	path length	connectivity	steps	Gini
baboon	25	1.73	6.03	514	0.121
chimp	23	1.71	5.61	509	0.132
karate club	34	2.42	2.17	192	0.176
coastal Agta	37	1.32	20.28	375	0.153
forest Agta	53	2.03	12.07	145	0.153
department	51	3.64	1.60	43	0.265

Prior research by Migliano *et al.* [32] theorized that the structure of hunter–gatherer networks may accelerate cumulative cultural evolution. Others, examining the transmission of social behaviours in chimpanzees, have argued that chimpanzee social systems are ‘pre-adapted’ for similar forms of cumulative culture [41]. In other words, it is possible that some social network structures may facilitate or impede the emergence of cumulative cultural evolution [4]. Here, when compared with two primate networks, hunter–gatherer networks outperform both in time to completion of the Potions Task and have a higher Gini. Given that crossover events in the model rely on subgroups of the population working on different parts of the global task, this partitioning via structure can be viewed as a temporary form of a division of labour. The explicit advancement of such specialization in networks can likely explain the difference in performance between Agta hunter–gatherers and academic departments. While it may be true that chimpanzee networks are pre-adapted for cultural transmission, a question worth asking is to what extent broader human social networks are pre-adapted for more recent phenomena like role specialization and cumulative cultural evolution [32,42].

## 3. Discussion

Our findings highlight how network structures which scaffold innovation and collective problem-solving also create inequality between individuals within networks. In our simulations, every factor which helped scaffold collective performance led to an opposite and proportional trend in the payoffs agents received (figure 3). Larger networks, less connected networks, more cliquish networks and networks which limited the diffusion of information all improved collective performance at these tasks but created unequal payoffs in the population.

Prior studies on collective problem-solving have proposed a number of mechanisms for bolstering a population’s collective intelligence [5,6,8,10,11,21,25,27–29,31,32,43]. These are often presented without consideration of tradeoffs between population-level performance at these tasks and the impact these factors have on individual agents, leading to the illusion that factors such as reduced connectivity and information transmission represent ‘a free lunch’ for populations, or at worst, merely sacrifice the time needed to reach high-quality solutions. This perspective is particularly prevalent in the economics of innovation where it has been proposed that technological change provides a cost-free benefit to groups [44]. Our results, that the unequal dispersion of benefits in groups which facilitate innovation, challenge the assumption that technological change comes cost-free. Instead, the cost is borne by unequal work within these groups. This may, in turn, have important social ramifications.

Our results also address the relationship between an individual’s productivity and their two forms of capital: human capital, broadly defined as an individual’s personal attributes such as skill level, intelligence or exploitable knowledge; and social capital, broadly defined as an individual’s network of relationships [45]. The commonly perceived tradeoff between these two points of emphasis in the social sciences have led to both agent-positive (those which emphasize individual behaviour) [46] and agent-negative (those which emphasize structural arrangements) [3] views of progress. An emphatic divide between these separate frameworks is why progress in the sciences and technologies appear to be facilitated by the appearance of geniuses. Is it simply the case that the secret to improving science is finding such geniuses in the general population or is it the case that structural factors facilitate such individuals to have overly proportional contributions to the growth of knowledge? Our results indicate some support towards factors facilitating the latter perspective, showing that ‘genius effects’ can arise in a population of entirely ‘dumb’ agents.

We additionally find three results particularly relevant to the study of collective behaviour and innovation which are orthogonal to our findings on inequality. First, that connected caveman networks perform better when the number of cliques are maximized and when clique sizes are minimized, speaks to the specific role of group division and composition in complex tasks, and implies that having many small groups may be better than having fewer large groups. Second, we find that populations of all sizes and clique compositions perform similarly *per capita* when the overall number of combinations are taken into account, even though larger networks possess a higher average degree at any given level of edge probability. This speaks strongly to the tradeoff between connectivity and scale in problem-solving networks. Finally, that random link alteration, as a form of inter-group communication, plays a limiting factor in innovation and stands in contrast to prior explorations of the phenomenon.

Several limitations of the current study should be noted. First, the Potions Task is limited in its ability to recover some of the earlier results on population dynamics and innovation in which the role that information *loss* plays is central. Similarly, while we could not find a positive relationship between link alteration and innovation, our analysis was limited to randomly rewiring network connections. It is possible that certain strategies for non-random, targeted link alterations could produce different effects, such as when those strategies involve substantial changes to network centrality. Second, although we extensively show how the relationship between an agent’s position in the network and the amount of work they do as one mechanism leading to ‘genius’ effects, the individual 'skill' of an agent is technically never taken into account—all of our agents have identical abilities. Third, the model ignores the mechanical processes involved in turning innovative ideas into innovate products, which are often costly. Thus, the model as it currently stands applies more readily to innovations that require minimal overhead, such as behaviours rather than complex technologies. To the extent that inequalities in knowledge are likely to correlate with inequalities in resource access, the nature of our results may be further exacerbated, as those late to acquire knowledge may find themselves without resources needed to put that knowledge to use. In other words, the relationship between inequality of solutions, which we measured in this study, and more salient forms of inequality in the real world such as inequality of outcome, opportunity, or resources are complex questions in the real world and not addressed by our model [13].

Our research indicates that properties of collective organization and communication that facilitate innovation also facilitate increased heterogeneity of work within these groups. More specifically, this heterogeneity indicates that even in a population consisting entirely of ‘dumb’ agents, ‘genius effects’ can arise in some agents rather than others. In the real world such inequality has been recognized as leading to more drastic effects in both performance and income, such as the Pareto principle or the ‘law of the vital few’ [14]. Future work on the economics of innovation and entrepreneurship should therefore attend more specifically to network-level effects which give rise to these phenomena and should ask to what extent crucial innovators play the role of information synthesizers or aggregators in their broader networks. Although our findings show that network factors which give rise to innovation also give rise to inequality of performance, further research on agent-level outcomes in network tasks is needed, and we suspect that future modelling work may require the development of more complex multi-task environments or introduction of agent-specific motivations.

## 4. Material and methods

Our model follows the approach of Migliano *et al.* [32] and Cantor *et al.* [20] in modelling the Potions Task from a prior online experiment [31], but generalized to support arbitrary network structures adding several dynamics such as dynamic link alteration and having agents adjust the probability that they share novel innovations with their connections. Here, we provide a description of the model below, written in Python using the Mesa library [47].

### (a) Entities and state variables

Each model is comprised of agents assembled as nodes on a network. The principle model dynamic is elaborated through pairs of agents (dyads) combining sets of items beginning from an initial inventory of six that each agent starts with. Each ideal network is unweighted, but several of the real-world networks (chimpanzee, baboon and Agta hunter–gatherer) are weighted networks.

Items in each agent’s inventory are initialized in an array containing three values: the name of the item, the item’s score and the item's rank. In order to craft new items, three specific items must be combined between two agents. With the initial set of six items, there are two valid combinations which can be made: a combination of items a1, a2 and a3 or a combination of items b1, b2 and b3. These will form items 1a and 1b, respectively, which can be combined with items from the initial set in order to make further items. Agents select each item based on a probability calculated by dividing each specific item’s score by the sum of the scores of all the items in the inventories. Because each novel item discovered is on another ‘tier’ above the set of items used to create it and has a higher score, this creates path dependency in the model (agents are unlikely to go back and use older items in their inventory over new ones). There are four such ‘tiers’ of items which can be discovered and combined and a fifth tier, which is formed by combining each of the two items on the two separate fourth tiers with one another. The specific scores and item combinations are seen in figure 1.

Each ideal network has a number of state variables which are manipulated. Random networks are initialized as Erdös–Rényi networks with the number of agents and critical edge probability as initial variables, ring networks are initialized with the number of agents as initial variables, and connected cavemen are initialized with the number of cliques and clique size as initial variables. Common to these network structures are the probability of diffusion (or the probability that each individual neighbour of an individual agent which discovers an item receives a new innovation when the focal agent discovers one) and the probability of link alteration, or the probability that each agent has one of its links removed and a new one added at the end of each step in the model.

### (b) Model process

Following initialization, the model runs through several steps where agents select a partner, select which item(s) from their inventory they will be combining with their partner, making a combination, and, if combinations are successful, diffusing it to their neighbours. These steps are as follows.

1. Model initialization: a network is created with its respective parameters. For each node of the graph, an agent is initialized with a score of zero and an inventory comprised of six items: three from an ‘A trajectory’ and three from a ‘B trajectory.’ Each item in the inventory is comprised of three parts: the name/level of the item (e.g. a1, a2, a3, b1, b2, b3), its rank, and a score which each initial item and items discovered thereafter carries for itself (with innovation values of 6, 8 and 10 for the three initial items in each trajectory).
2. Dyad selection: at each step, each agent chooses a partner they are connected to on the network with a random probability. In weighted networks, this probability is non-random and is calculated as each edge weight and agent has divided by the sum of all of its weights. As neighbours are simply chosen with some probability, it is possible for a focal neighbour to select an individual which is already interacting with them (e.g. if a network is initialized with just two agents, the two agents will simply select each other).
3. Item selection: in the model, new items are formed by triad combinations of old items. As triad combinations are made between dyads of agents, the focal agent randomly selects whether it will be combining either one item or two items with their partner, who provides the remainder. The focal agent and its partner then cycle through their respective inventories, assigning probabilities to each item in the array. This is obtained by summing the innovation scores of each item and dividing individual scores by each sum (e.g. the initial inventory innovation scores of 6, 8, 10, 6, 8, 10 will yield respective probabilities of 0.125, 0.167, 0.208, 0.125, 0.167, 0.208).
4. Item combination: agents and their partners then select the number of items previously assigned to them in the last step, based on items’ calculated probabilities without replacement, and combine their items. The combination is saved as a list and compared with lists of valid combinations copied directly from Derex & Boyd [31] (figure 1). If an invalid combination is made, nothing happens. If a valid combination is made, then the agent and their partner add a new innovation (with its own respective innovation values, rank and scores) to their inventories.
5. Innovation diffusion: if a new innovation is added to the agents’ inventories, both agents then check the inventories of all of their partners and spread it to neighbours which do not already possess it with some probability of diffusion. This probability is calculated per edge, such that an agent with a probability of 0.5 diffusion will diffuse the innovation to any of its individual neighbours at a 50% chance. In a fully connected network with a full probability of diffusion, the entire network obtains the innovation; with a 0.5 probability of diffusion, approximately half the network will acquire the innovation.
6. Scoring: scores are then obtained for each agent based on the tier of discovery an agent has obtained: with the first tier yielding a score of 48, second tier 109, third tier 188 and the fourth tier (which requires a crossover from the A and B trajectory) being 358. The maximum score of an item in an agent’s list is determined to be their overall score.
7. Connection alteration: at the end of each step, each network can rewire its connections. With some probability between 0 and 1, each agent randomly selects a partner they are connected to, removes its link from that partner and adds a link with a partner they were previously unconnected to. At a probability of 1, all agents will change partners; with a probability of 0.5, half of the network will change partners.
8. End and crossover: the simulation ends either when the network has achieved a ‘crossover event’, whereby the final inventions in both the A trajectory and the B trajectory are themselves finally combined, indicating the network has discovered and united both paths of exploration, or when it has reached 1000 steps (when the majority of networks >15 individuals will have already obtained a crossover event. For a list of success rates at 1000 steps, see electronic supplementary material, appendix, table S2).

### (c) Data collection

Data were collected at the end of each step in the model. Agent-level data include each agent’s score and its inventory. From this, an average score, a Gini coefficient and the maximum score of all the agents were collected. Simulations ended when any agent achieved a maximum score of 358, indicating that a crossover event had been accomplished. The step at which the crossover event had taken place was then recorded.

The Gini coefficient is a measure of inequality based on the mean of absolute differences between all pairs of individuals in the population [15]. The Gini coefficient is defined as

4.1 $$\frac{\sum_{i=1}^{n}\sum_{\;j=1}^{n}|x_{i}-x_{j}|}{2n^{2}\bar{x}},$$

where *n* is the number of agents in the population and *x* is the value of an individual agent’s maximum item score.

Using NetworkX [48], we additionally recorded some summary statistics about each of the networks including the network’s initial and final path length, its initial and final clustering coefficient, and whether the network was a complete network at initialization. For several arrangements of the connected caveman (electronic supplementary material, appendix, table S1) and real-world networks (table 1), we also calculated the average degree of the network.

## Data Availability

A GitHub repository containing code for all simulations, the edge lists of the real-world networks, and the code for analyses has been deposited in Zenodo: https://doi.org/10.5281/zenodo.10037521 [49]. Data used in the analyses are stored in a separate Dryad repository: https://doi.org/10.5061/dryad.hhmgqnknz [50]. Supplementary material is available online [51].
